# Ultrahigh-Q optomechanical crystal cavities fabricated in a CMOS foundry

**DOI:** 10.1038/s41598-017-02515-4

**Published:** 2017-05-30

**Authors:** Rodrigo Benevides, Felipe G. S. Santos, Gustavo O. Luiz, Gustavo S. Wiederhecker, Thiago P. Mayer Alegre

**Affiliations:** Applied Physics Department, Gleb Wataghin Physics Institute, University of Campinas, Campinas, 13083-859 SP Brazil

## Abstract

Photonic crystals use periodic structures to create frequency regions where the optical wave propagation is forbidden, which allows the creation and integration of complex optical functionalities in small footprint devices. Such strategy has also been successfully applied to confine mechanical waves and to explore their interaction with light in the so-called optomechanical cavities. Because of their challenging design, these cavities are traditionally fabricated using dedicated high-resolution electron-beam lithography tools that are inherently slow, limiting this solution to small-scale or research applications. Here we show how to overcome this problem by using a deep-UV photolithography process to fabricate optomechanical crystals in a commercial CMOS foundry. We show that a careful design of the photonic crystals can withstand the limitations of the photolithography process, producing cavities with measured intrinsic optical quality factors as high as *Q*_*i*_ = (1.21 ± 0.02) × 10^6^. Optomechanical crystals are also created using phononic crystals to tightly confine the GHz sound waves within the optical cavity, resulting in a measured vacuum optomechanical coupling rate of *g*_0_ = 2*π* × (91 ± 4) kHz. Efficient sideband cooling and amplification are also demonstrated since these cavities are in the resolved sideband regime. Further improvements in the design and fabrication process suggest that commercial foundry-based optomechanical cavities could be used for quantum ground-state cooling.

## Introduction

Shortly after the first demonstrations of bandgap structures, both at microwave^1^ and infrared frequencies^2^, photonic crystal structures have emerged as strong candidates for applications in optical and radio-frequency (RF) circuits. In particular, the realization of ultra-high optical quality factors^3, 4^ allowed for several applications, such as ultra-small filters^5^, fine-tuned demultiplexing devices^6^ and high-sensibility sensors^7^. Additionally, their mode volume smaller than a cubic wavelength^8^ leads to strong light-matter interaction, in which optical nonlinear regimes can be easily accessed^9, 10^. Properly designed photonic crystal cavities (PhCs) may also be used to support localized long-living mechanical modes that can efficiently interact with the optical field^11, 12^. This optomechanical interaction has been used not only to study the fundamentals of quantum interaction between light and matter^13, 14^ but also to develop devices for applications^15^, such as RF-memories^16^, accelerometers^17^, torque sensors^18^ and synchronization of mechanical oscillators^19^. However, most of these devices and applications rely on traditional high-resolution electron beam lithography fabrication, a process that is inherently slow, limiting this solution to small-scale applications or research. On the other hand, complementary metal-oxide-semiconductor (CMOS) fabricated structures have proven to be a mature and viable option for photonic^20–23^ and optomechanical^24^ structures.

Here we take a step forward on the CMOS foundry integration approach by designing and fabricating a 2D-optomechanical crystal. We show that it is possible to fabricate a photonic crystal cavity with intrinsic optical quality factors as high as *Q*_*i*_ = 1.21 × 10^6^, arguably the largest ever reported based on optical lithography. Embedding this optical cavity within a phononic shield^12^ resulted in optomechanical crystals where several mechanical modes with frequencies around Ω_*m*_ = 2*π* × 2 GHz are demonstrated, with coupling rates as large as *g*_0_ = 2*π* × 91 kHz. Finally, the increase in the mechanical quality factor due to the phononic shield allowed us to demonstrate cooling and amplification of the mechanical mode at low temperature. Our results pave the way for CMOS foundry based optomechanical crystals as building blocks for fundamental studies and applications.

## Photonic crystal cavities

**Figure 1:**
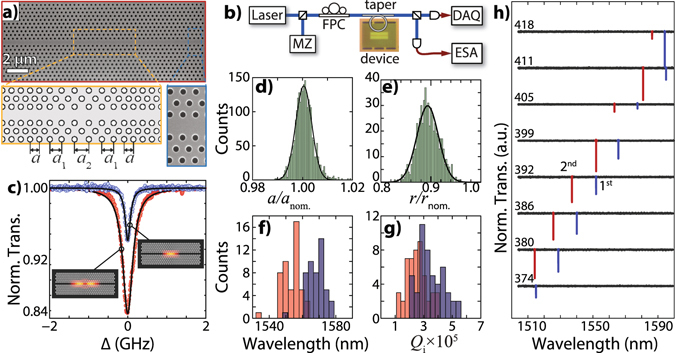
Photonic crystals (**a**) SEM (scanning electron microscope) image of a typical photonic crystal cavity (PhC) with a zoomed image showing the uniformity of the holes. The magnified schematic shows the design of the PhC with the silicon-defect waveguide. Around the center of the cavity, the lattice parameter is changed to confine light. The nominal sizes are *a* = 410 nm, *a*_1_ = 420 nm and *a*_2_ = 430 nm, hole radius of *r* = 117 nm and slab thickness of *t* = 220 nm. (**b**) Highest optical quality factor observed for the fundamental (blue − *Q*_*i*_ = 1.21 × 10^6^) and second order (red − *Q*_*i*_ = 6.93 × 10^5^) optical modes, respectively. The insets show finite element simulations (FEM) of the intensity profile for both modes. (**c**) Schematic of the optical setup used for optical and mechanical characterization. A tunable laser is monitored by a Mach-Zehnder (MZ) interferometer while its radiation is evanescently coupled to the device using a tapered optical fiber, after passing through a fiber polarization controller (FPC). The optical transmission is measured using a slow photodetector (PD) coupled to a data acquisition card (DAQ), while a high-bandwidth PD connected to an electrical spectrum analyzer (ESA) is used to characterize the rapid modulation impinged on the signal by the mechanical oscillation. Distribution of the lattice parameter (**d**) and hole radius size (**e**) based on high-resolution SEM images. Both values are normalized by their nominal sizes *a*_nom._ = 410 nm and *r*_nom._ = 117 nm (see Supplementary Material). Central wavelength (**f**) and intrinsic optical quality factor (**g**) measured for the same cavities used to produce the distributions seen on (**d**) and (**e**). The blue (red) bars represent the first (second) order optical mode. (**h**) Broadband optical transmission spectra for cavities with fixed filling factor (*r*/*a* = 0.285) and lattice parameters from *a* = 374 nm to *a* = 418 nm. The first and second order optical modes are labeled by the blue and red colors respectively.

One of the main constraints for the realization of optomechanical crystals based on commercial CMOS foundries are the design rules imposed by these facilities. In order to meet the foundry fabrication requirements, we have designed a modified version of a photonic crystal cavity based on a previous work^4^. The device consisted of a hexagonal lattice photonic crystal with a line-defect waveguide, and a modification in lattice parameter at the central region to form an optical cavity, as shown in Fig. 1a. Our approach was to create a deeper optical defect (see Supplementary Material), where the lattice parameter change was larger than previously reported^4^. We show that this deeper defect can efficiently confine photons, improving the optical quality factor when compared to previous photonic crystal cavities fabricated by optical lithography^20, 22^.

To fully characterize these cavities we performed a statistical analysis of their final geometrical and optical properties. The position and radii distribution of the PhC holes were determined from several high-resolution scanning electron microscope (SEM) images – obtained from nominally identical cavities –, as the one shown in Fig. 1a. The devices were probed by a tunable laser that is evanescently coupled to the cavity using a tapered optical fiber, as shown in Fig. 1b. A Lorentzian fitted to the DC optical transmission spectrum, Fig. 1c, yields both the resonant wavelength and linewidth of the cavities.

Since the thickness of the slab is fixed (*t* = 220 nm), a given lattice parameter *a* defines the central position of the photonic bandgap, whereas the ratio between the hole radius and the lattice parameter, *r*/*a*, determines the bandgap width. Figure 1d,e show histograms of the lattice parameter *a* and hole radius *r* measured from the SEM images, as discussed in Supplementary Material. As we are only interested in the standard deviation of each parameter, as well as the ratio between them (*r*/*a*), we rescaled each SEM image to match the nominal lattice parameter.

A remarkable precision of the lattice parameter is observed with a standard deviation smaller than 1%. This is corroborated by the measured dispersion of the optical resonant wavelength for both - first (blue) and second order (red) - modes, as shown in Fig. 1f. On the other hand, the statistics for the hole radii have two important features. The holes are consistently smaller than their nominal radii (10% smaller) and have a larger standard deviation (5%) when compared to the lattice parameter. The smaller hole radius could be easily compensated by simply changing the initial hole radius size, or using a different lithography dose. The standard deviation, however, translates into a disorder in the lattice bands that directly affects the optical confinement and could lead to a smaller optical quality factor. As a result, the measured intrinsic optical quality factors of these devices, for both the first (blue) and second order (red) optical modes, have a broader distribution than the resonant wavelength, as shown in Fig. 1g. However, they are still centered on a value of *Q*_*i*_ = 3 × 10^5^, which is larger than previously reported similar devices also fabricated by optical lithography^22, 23^.

Besides the high optical quality factor, another important feature is the ability to design and predict the resonant wavelength of the optical cavities. In order to evaluate this possibility, we have fabricated – on the same chip – several slightly different cavities with lattice parameters and hole radii changing a few percent around a central value (*a* = 400 nm *r* = 114 nm), while keeping a fixed filling factor *r*/*a* = 0.285. As a result, we would expect roughly a 2.5% change of the cavities’ resonant wavelengths. The measurements are shown in Fig. 1h, where we can observe a steady increase in the resonant wavelengths for larger lattice parameters, as expected. Conversely, fine tuning the radius, while keeping the lattice constant fixed, allows achieving a desired filling factor. This have a direct impact in the optical quality factors as seen in Fig. 1b, where we show the largest measured optical quality factors for the first and second order optical modes. The measured intrinsic optical quality factor for the first order mode is as high as *Q*_*i*_ = (1.21 ± 0.02) × 10^6^, for a cavity with nominal values of *r* = 121 nm and *a* = 405 nm, showing that a careful design of photonic crystals can withstand the limitations of the photolithography process and result in ultra-high quality optical cavities.

## Optomechanical crystals

**Figure 2:**
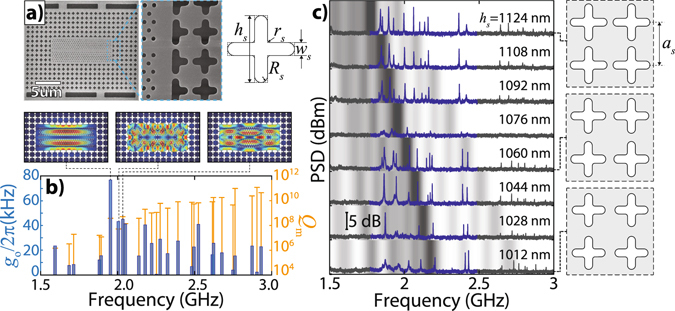
Optomechanical crystals (**a**) SEM images of a fabricated shielded cavity. The inset highlights the rounded shape acquired by the cross holes. The geometrical parameter definitions of the phononic shield are depicted. (**b**) FEM for the optomechanical coupling (blue bars, left scale) and mechanical quality factors (yellow lines, right scale) for the PhC surrounded by a shield with values of *a*_*s*_ = 1265 nm, *h*_*s*_ = 1125 nm, *w*_*s*_ = 300 nm. We observe a logarithmic improvement in quality factors for modes inside the mechanical bandgap, which should be compared to the respective optomechanical coupling rates of each mode. The insets show the mechanical mode displacement profile for the three largest optomechanical coupling rates. The simulation only takes into account mechanical modes symmetric with respect to the central plane of the slab. (**c**) Mechanical spectra for eight different mechanical structures, whose crosses lengths (*h*_*s*_) are indicated above the spectra. The shading behind them is proportional to the density of states for each structure, based on unit-cell FEM simulations. Darker (lighter) regions correspond to a higher (lower) density of states. The simulations parameters were *w*_*s*_ = 297.4 nm, *R*_*s*_ = 148.7 nm and *r*_*s*_ = 29.74 nm and a changing *h*_*s*_, shown in every spectrum.

To pursue a quasi-2D optomechanical crystal, we fabricated a set of PhC embedded into a phononic shield based on cross-shaped holes (Fig. 2a). The phononic shield enhances both mechanical quality factors and optomechanical coupling rates, thus allowing the measurement of the mechanical modes of the photonic crystal cavity slab^12^. The cross-shaped phononic crystal is designed to have an acoustic bandgap for the in-plane modes with frequencies between 1.5 GHz and 4.0 GHz. Performing FEM simulations we calculated the optomechanical coupling rate *g*_0_ and the radiation limited mechanical quality factor (see Supplemental Material for details), using a shield lattice parameter of *a*_*s*_ = 1265 nm, *h*_*s*_ = 1125 nm, *w*_*s*_ = 300 nm (see Fig. 2a for geometrical definitions) and the nominal values of the PhC cavity shown in Fig. 1a. Following a perturbation theory approach, the optomechanical coupling rate can be calculated as ${g}_{0}=-\,(\omega \mathrm{/2)}\langle \vec{E}|{\rm{\Delta }}\varepsilon |\vec{E}\rangle /\langle \vec{E}|\varepsilon |\vec{E}\rangle$, where the inner products ratio between the unperturbed electric field distribution ($\vec{E}$) measures how much the optical mode resonance frequency, *ω*, varies when the dielectric constant changes from *ε* to *ε* + Δ*ε* due to the action of the mechanical mode. Strain-induced modifications of the refractive index (photo-elastic effect)^11^ and boundaries deformation^25^ are both taken into account when evaluating Δ*ε*. The phononic shield confines the mechanical modes within the optical photonic crystal cavity slab resulting in simulated optomechanical coupling rates as large as *g*_0_ = 2*π* × (76 ± 4) kHz (Fig. 2b).

However, as shown in the detail of Fig. 2a, the fabricated crosses have rounded corners that slightly changed the mechanical bandgap. To evaluate the role of the phononic shield we designed and tested several nominally identical optical cavities embedded in a cross-shaped phononic crystal with different acoustic shield cross lengths (*h*_*s*_). The devices are once again probed by a tunable laser that is evanescently coupled to the cavity through a tapered optical fiber resting on the cavity. The typical intrinsic optical quality factor of these cavities is *Q*_*i*_ = 2.2 × 10^5^ (loaded *κ* = 2*π* × 1.4 GHz). The phase of the intra-cavity optical field is modulated by the thermally excited mechanical modes. The cavity response then converts the phase into amplitude modulation, which is measured using a fast detector and an electrical spectral analyzer (ESA) to determine both the mechanical frequency and linewidth.

The measured mechanical spectra for samples with varying sizes of the cross length (*h*_*s*_), while keeping the other parameters constant, are shown in Fig. 2c. We choose to vary *h*_*s*_ since this sole parameters is enough to modify the mechanical bandgap size^26^. In order to take into account the modified geometry, we use FEM simulations to calculate the quasi-2D in-plane phononic bandgap, using the geometric parameters extracted from SEM images. The results are translated into a density of states (DOS) map, superimposed by the mechanical spectra, where darker (lighter) shading refers to higher (lower) DOS. The mechanical modes follow the trend of the FEM simulations, having higher mechanical quality factors (narrower linewidth) for modes within frequency regions of lower DOS (lighter regions). The measured spectra reveal multiple modes above 2 GHz that, associated with the narrow optical linewidths, put these modes in the so-called resolved sideband regime (*κ* ≤ Ω_m_), where efficient cooling and amplification of the mechanical mode can take place.

In order to achieve enhanced optomechanical interaction, along with the resolved sideband regime, it is often desirable to have a low mechanical loss in optomechanical cavities. At room temperature, typical mechanical quality factors were *Q*_*m*_ = 2000 for modes within the calculated bandgap. Two main loss mechanisms contribute for this modest *Q*_*m*_-factor: the clamping loss associated with imperfections in the phononic shield and phonon-phonon scattering losses (Landau-Rumer) mediated by temperature^12^. While the former is limited by the phononic structure including fabrication imperfections, the latter should be drastically reduced at low temperatures.

In order to reveal the influence of the clamping loss term, we have cooled down the sample using a continuous flow liquid helium cryostat to drastically reduce the phonon-phonon scattering. During the measurement period, the sample temperature was kept at 25 K - temperature measured using a calibrated silicon sensor attached to the sample holder. At low temperatures, the mechanical losses decreased to *γ*_*i*_ = 2*π* × 150 kHz for a mode at Ω_m_ = 2*π* × 1.89 GHz, enabling the observation of optomechanical cooling and heating^27^ of the mechanical mode. Using the setup of Fig. 1c, we scanned the frequency of a tunable laser and simultaneously recorded the mechanical power spectrum density (PSD) and the optical transmission. The laser frequency scanning rate (0.1 Hz) was intentionally kept much lower than the RF spectrum analyzer acquisition rate (33 Hz).

**Figure 3:**
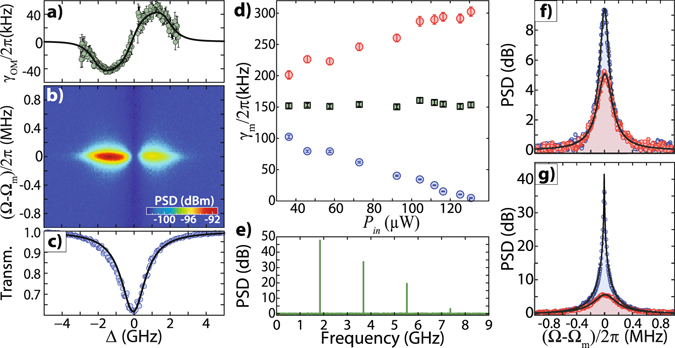
Back-action measurements (**a**) Optomechanical linewidth modification as a function of the laser detuning for the mechanical mode in Ω_m_= 2*π* × 1.89 GHz. The points were obtained by fitting a Lorentzian to each mechanical spectra shown in (**b**). The black line indicates the fitting of the theoretical model. (**b**) Mapping of the mechanical mode in Ω_m_= 2*π* × 1.89 GHz, as a function of the laser detuning around an optical resonance. (**c)** Optical mode used for the sweep in (**a,b**), with *Q*_*i*_ = 1.2 × 10^5^. Each point in this spectrum corresponds to a mechanical spectrum in (**b**) and a fitting point in (**a**). (**d**) The extreme (maxima or minima) effective mechanical linewidth as a function of input power in the cavity. The blue and red dots represent the mechanical linewidth for the laser at the blue (Δ < 0) and red sides (Δ > 0). The green squares are an average of both measurements which gives the intrinsic mechanical linewidth. (**e**) Mechanical spectrum for the cavity in the regime of self-sustained oscillation. The cavity is induced to oscillate in just one mode (Ω_m_= 2*π* × 1.89 GHz), with very high signal and narrower linewidth. An RF frequency comb with frequencies up to 7.5 GHz is generated. (**f**) Mechanical mode for a low input power *P*_*in*_= 36.4 *μW*, for the laser blue and red detuned, with respective resonance colors. Back-action can already be observed. (**g**) Increasing the input power up to *P*_*in*_= 146.5 *μW*, we see a ∼35 dB difference between blue and red curves and a remarkable change in linewidth.

The results for the lowest input power of 36.8 *μ*W are shown in Fig. 3a–c. When the laser is tuned to the blue side of the optical resonance (Δ = *ω*_*l*_ − *ω*_0_ < 0), the mechanical motion is amplified, as indicated by the noticeable PSD increase in the color map of Fig. 3b. This is confirmed by modification of the mechanical linewidth (*γ*_*m*_ = *γ*_*i*_ + *γ*_OM_) as a function of the laser detuning. Fig. 3a shows such modification of the optomechanical damping rate (*γ*_OM_), from which we extracted an optomechanical coupling rate of *g*_0_ = 2*π* × (91 ± 4) kHz and an intrinsic mechanical linewidth of *γ*_*i*_ = 2*π* × (148 ± 2) kHz. We also explored the relation between the optomechanical damping rate and the optical input power in Fig. 3d, where the mechanical linewidth for both blue and red detuned laser from the cavity are shown. Figure 3f and 3g show the measured and fitted mechanical spectra for the lowest and largest optical input powers, respectively; the blue and red curves correspond to a laser-cavity detuning matching the mechanical frequency (Δ = ±Ω_*m*_).

Above a power threshold (*P*_*in*_ = 130 *μ*W in our case), the mechanical mode undergoes a Hopf bifurcation and reaches a self-sustained oscillation regime^27^. Further increasing the optical input power generates large enough mechanical oscillation to enter a non-linear regime where an RF frequency comb is generated with frequencies up to 7.5 GHz, as shown in Fig. 3e. Reaching self-sustained oscillation regime clearly demonstrates that the high optomechanical cooperativity $C={n}_{{\rm{cav}}}{g}_{0}^{2}/\kappa \gamma  > 1$ (a metric for an optomechanical system) limit can be realized in these CMOS-Foundry compatible devices (*n*_cav_ being the intracavity photon number).

## Conclusion

In summary, we have designed a photonic crystal cavity resilient to the limitations of the photolithography process used in a commercial foundry. As a result, we could measure devices with ultra-high Q factors on the order of 10^6^. We have also shown how a mechanical crystal can be fabricated in a way to create a quasi-2D optomechanical crystal cavity where low mechanical damping loss can be obtained. Finally, low-temperature measurements revealed the possibility of using CMOS fabricated structures for efficient back-action measurements. Further improvement in the mechanical shield, along with the presently demonstrated ultra-high optical quality factor, could easily drop the single photon cooperativity by two orders of magnitude, thus enabling quantum ground-state cooling in these devices.

## Methods

### Fabrication

The devices were fabricated through the EpiXfab initiative at IMEC on a silicon-on-insulator wafer (top silicon layer of 220 nm over 2 *μ*m of buried silicon oxide). The holes and crosses structures were both designed in the same layer. In each optical and optomechanical cavity, the holes were referenced to a single hole object in the GDS design. Our optical defect depths were designed considering possible mismatch between our design and foundry grids. More details about the fabrication are presented in the Supplementary Material.

An in-house post-process step was performed to selectively and isotropically remove the buried oxide using a buffered solution of diluted hydrofluoric acid (HF + H_2_O 1:8) in order to mechanically release the structures. Finally, a piranha (H_2_SO_4_ + H_2_O_2_ 3:1 @ 140 °C) cleaning step to remove any organic residues followed by an HF dip (HF + H_2_O 1:10) was performed, which resulted in an consistent increase of the measured optical quality factors.

### Data Availability

The datasets generated and/or analysed during the current study are available from the corresponding author on reasonable request.

## Electronic supplementary material

Supplemental Materials: Ultrahigh-Q optomechanical crystal cavities fabricated in a CMOS foundry
