# Supplementary material for: Ultrahigh-Q optomechanical crystal cavities fabricated in a CMOS foundry
Source: Sci Rep. 2017 May 30;7:2491. doi: 10.1038/s41598-017-02515-4 (PMC5449385; doi:10.1038/s41598-017-02515-4)
Supplement: Supplementary file 1 — Supplemental Materials: Ultrahigh-Q optomechanical crystal cavities fabricated in a CMOS foundry [file 41598_2017_2515_MOESM1_ESM.pdf]

Supplemental Materials: Ultrahigh-Q optomechanical crystal cavities fabricated in a CMOS foundry

Rodrigo Benevides, Felipe G. S. Santos, Gustavo de O. Luiz, Gustavo S. Wiederhecker and Thiago P. Mayer Alegre
Applied Physics Department, “Gleb Wataghin” Physics Institute, University of Campinas, Campinas 13083-859, SP, Brazil

Lattice parameter and hole size statistics

The information about size of holes (radius r) and their distances (lattice parameter a) were obtained from high resolution (2048×1887 px) SEM images. The scanned areas were 3.7×3.4 μm^2, which renders the SEM pictures a resolution of ~ 3.2 nm^2 per pixel. An image processing algorithm was used to fit each hole to a circle as shown in the figure S1**a)**. The fitting results for the holes center coordinates (x_i,y_i) were used to determine the relative distances between holes $a_{i,j} = \sqrt{(x_i - x_j)^2 + (y_i - y_j)^2}$, which were used as the lattice parameters for each hole pair. The fitting also returned the radius (r_i) of each hole.

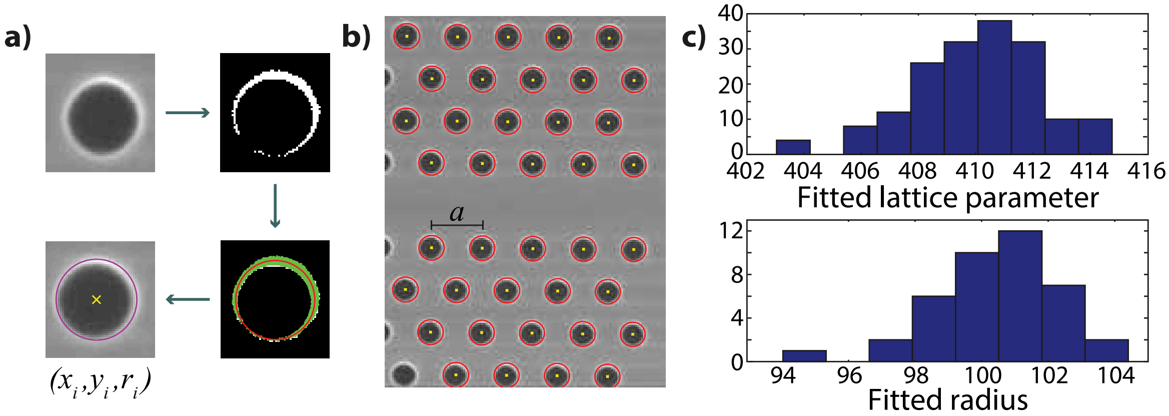

Figure S1. SEM-fitting example a) The process of fitting consists in modifying the image to a black and white picture, based on a given threshold related to the brightness of the original image. A circle is then fitted to the image, providing a center position and a radius. **b)** This process is made for every hole in the picture, resulting in an array of fitted circles. **c)** With the position of the circles’ centres, we obtain a distribution of distances (top), along with the fitted distribution of radii (bottom).

The absolute values are taken relative to the averaged measured value for lattice parameters, avoiding possible distortion effects due to astigmatism in the electron microscope. In Fig. S1**b)** we show the resulted fitting for a typical image. All images are taken far from the optical defect region, ensuring that the nominal values are retrieved. For this particular picture, we have a total of 39 fitted holes, resulting in 172 distances. The nominal values for this crystal were $a_{\text{nom}} = 410$ nm and $r_{\text{nom}} = 117$ nm. This process was repeated for each one of the 64 nominally identical cavities to obtain the histograms of Figs. 1**d-e** of the main text.

Based on our fitting results, our errors were smaller than 2 pixels, ensuring an uncertainty $< 4nm$. All optical measurements were done prior to the SEM images to avoid any contamination from the imaging process.

Photonic crystal geometry

The photonic crystal geometry is based on a 2D-hexagonal lattice, as shown in Fig. S2**a)**, where it is possible to see how the optical cavity is generated in the central region of the slab, using a modification of the lattice parameter. The modification of the holes distances is done in only one direction, such that the effective index of refraction changes smoothly in one axis (parallel to the waveguide) and it remains constant in the other direction.

In Fig. S2**b)**, we see how the change in the lattice parameter is done, in a continuous way along 10 unit cells, to avoid abrupt changes in the effective index of refraction that could lead to photon losses.

We also remove a line of holes, creating a line defect in the photonic crystal, as shown in Fig. S2**c)**. The bandgap of photonic crystal avoids the propagation of certain wavelengths inside the crystal, creating a waveguide in this line defect. Propagation in the out-of-the-plane direction is avoided for total internal reflection.

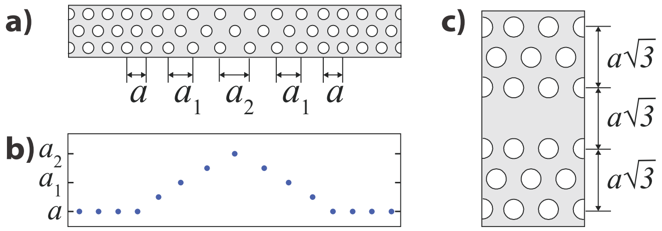

Figure S2. Geometry of the photonic crystal a) A change in the central region of the crystal generates photon confinement. **b)** The change is made smoothly, in an approximately gaussian defect. **c)** A line defect waveguide is created to guide light into the cavity.

Foundry grid snapping

When designing a device to be fabricated by a foundry, care must be taken when dealing with the grid size that will be used. Depending on the limiting size of the structure and the grid used by the foundry, strong modifications in the expected design can be obtained.

In our design, we have analyzed ways to improve photon confinement, matching the foundry limitation. A grid of 5 nm was used in the foundry fabrication, forbidding the possibility of performing a change in parameters smaller than it. Taking this limitation into account, we have designed a deeper defect than previously reported, such that we could match better the grid condition. In Fig. S3**a**, we show the difference between our design and a design based on a previous work[1]. Using a deeper defect allowed us to snap the holes' centres into the foundry grid. Conversely, the use of a shallower defect leads to an undesired snapping of the holes to different positions. We believe that this difference can change significantly photon confinement, as the shallower defect is not adiabatic.

However, even though we have taken care of it, the use of a hexagonal lattice forbids the complete matching between grid and design. In Figs. S3**b**-**c**, it is possible to see the mismatch effect along the 2D lattice for the two cases. We see that there is a higher disorder for a shallower defect when compared to ours. In particular, we have a complete matching in first row of holes, repeated in all odd lines from the waveguide. This corroborates our high quality factor results and highlights the importance of considering the mismatch in foundry designs.

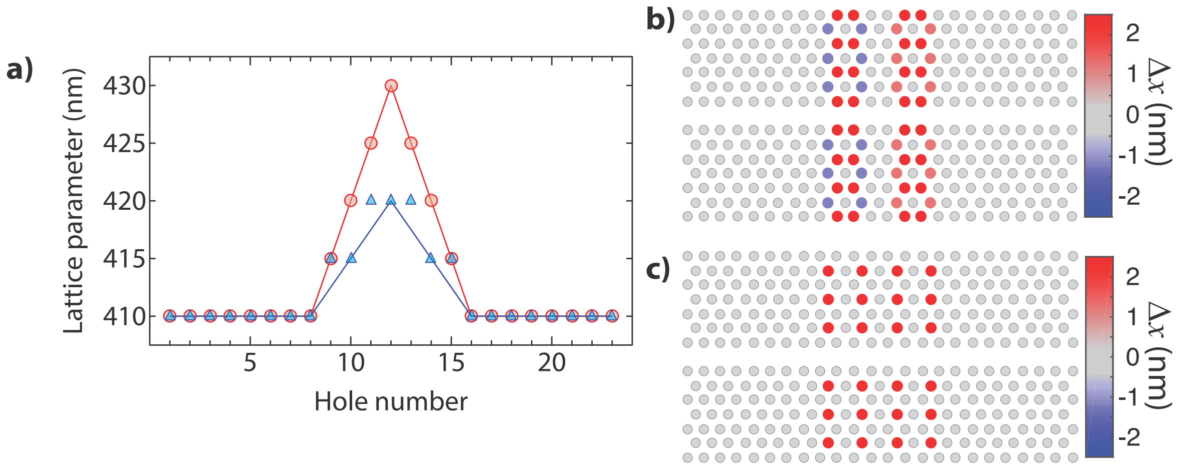

Figure S3. Comparison between shallower and deeper defects a) Red circles (blue squares) are the obtained lattice parameter due to foundry grid in the deeper (shallower) defect. Solid lines are the designed lattice parameter for each hole. **b)-c)** Difference along the waveguide direction (x-direction) between the lattice parameters for the grid snapped and as designed photonic crystal cavity for the deeper and shallower defects respectively. While for the deeper defect design the first rows around the waveguide are intact, several dislocation are expected in the same region for the shallower defect design.

Cross-section of photonic crystals

To evaluate the uniformity of the foundry etching process as well as its anisotropy, we present, in Fig. S4, cross-section images for the optical cavities. These images show smooth surfaces along the holes. Moreover, we can see a small inclination of the holes' walls ($\gtrsim 87^{\circ}$). This implies a change of the holes' radii of ± 4 nm around an average value. We estimate that this change in radius implies a change in less than 2% in the central wavelength of the modes, based on measurements performed (not shown) similar to those shown in Fig. 1**h** of the main text, but for varying radii.

Based on the microscope scale, we can estimate the real size of both lattice parameter and diameter of the holes. The cavity in the Fig. S4**a** has nominal sizes of $a_{\mathrm{nom}} = 402$ nm and $2r_{\mathrm{nom}} = 226$ nm. From the

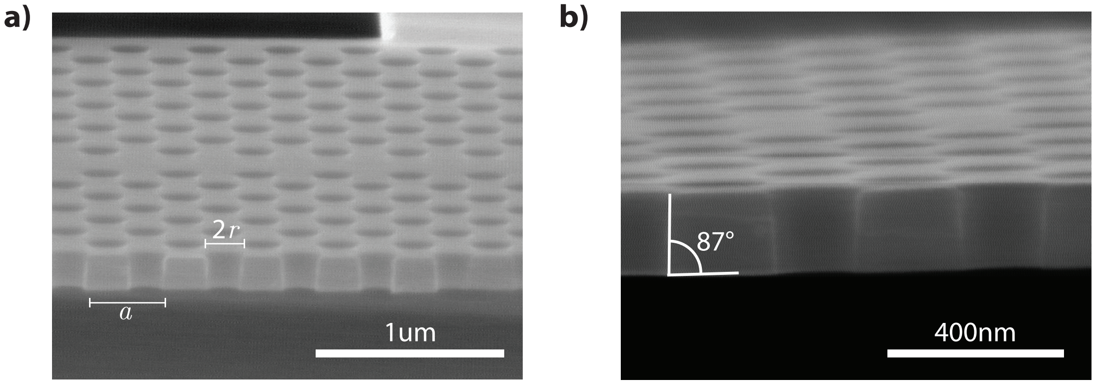

Figure S4. Photonic crystal cross-sections a) General vision of the photonic crystal cross-section. **b)** Hole wall angle measurement.

figure, we have obtained $a = 405$ nm and $2r = 203$ nm, where the radius is again smaller than expected, as it was already pointed out in the main text.

GDS layout

In this section, we present the layout used to fabricate our devices. For the cavities presented in the Fig. 1**c** and 1**h** of the main text, we have used the array shown in Fig. S5**a**. This array consists of single optical cavities where both the hole radius and the filling factor were changed in order to tune the resonant frequency. The radius changes from r = 105 nm to r = 129 nm and for a given radius (line) the filling factor changes from r/a = 0.266 to r/a = 0.301.

For the cavities presented in the Fig. 2 and Fig. 3 of the main text, we have used the array shown in Fig. S5**b**. The optical cavity for these devices are nominally identical ($a = 410$ nm and $r = 117$ nm), while we have changed the acoustic shield to study its role in mechanical modes. Each column in the layout corresponds to a cross width, ranging from $w_s = 180$ nm to $w_s = 220$ nm. The lines correspond to distinct cross lengths, from $h_s = 668$ nm to $h_s = 784$ nm. This array of cavities was used to generate the statistical analysis presented in Fig. 1**d-g** of the main text.

We provide downloadable versions for both designs as .GDS files in our web server[2,3].

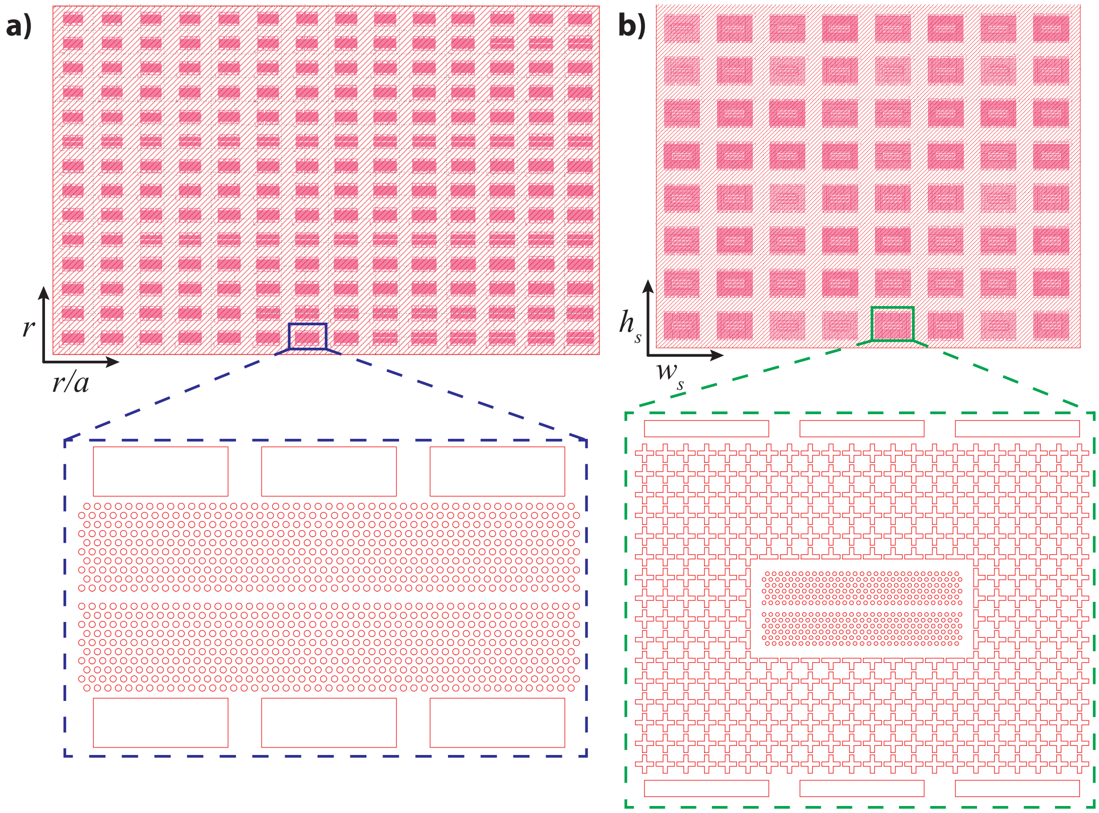

Figure S5. GDS layout a) Matrix used to study photonic crystal cavities. The arrows indicate the direction of increase for r and r/a. **b)** Matrix used to study optomechanical crystal cavities. The arrows indicates the direction of increase for h_s and w_s.

Perfectly matched layer parameters in FEM simulations

When simulating the optomechanical crystal mechanical radiation loss (Fig. 2**b** of the main text), we used a cartesian perfectly matched layer (PML) surrounding the phononic crystal device. Inside the PML, the coordinates are stretched in up to three directions according to the polynomial stretching function:

$$f_p(\xi) = s\lambda\xi^p(1-i) \tag{1}$$

where ξ is a dimensionless coordinate varying between 0 (close to the phononic crystal) to 1 (at the end of the computational domain); $\lambda = 3.6$ μm is the typical mechanical wavelength, $s = 3$ a scale factor, $p = 1.2$ the curvature parameter. The Q-factor is then obtained from the eigenfrequency Ω as $Q_m = Re[\Omega]/2Im[\Omega]$.

References

1. Sekoguchi, H., Takahashi, Y., Asano, T. & Noda, S. Photonic crystal nanocavity with a Q-factor of 9 million. *Optics Express* **22**, 916–924 (2014).

2. Benevides, R. S., Santos, F. G. S., Luiz, G. O., Wiederhecker, G. S. & Alegre, T. P. M. Photonic Crystal Cavity Design. URL http://www.ifi.unicamp.br/~gustavo/phc_cavity.GDS.

3. Benevides, R. S., Santos, F. G. S., Luiz, G. O., Wiederhecker, G. S. & Alegre, T. P. M. Optomechanical crystal cavity design. URL http://www.ifi.unicamp.br/~gustavo/optomechanical_crystal.GDS.
